# Prevalence of deleterious variants in cardiomyopathy genes in early-onset atrial fibrillation

**DOI:** 10.21203/rs.3.rs-7971912/v1

**Published:** 2025-11-19

**Authors:** Pia Lundegaard, Oliver Vad, Quim Vilaseca, Astrid Beyer, Christian Paludan-Müller, Laura Andreasen, Jesper Svendsen

**Affiliations:** University of Copenhagen; Rigshospitalet - Copenhagen University Hospital; Rigshospitalet - Copenhagen University Hospital; Rigshospitalet - Copenhagen University Hospital; Rigshospitalet - Copenhagen University Hospital; Department of Cardiology, Copenhagen University Hospital Rigshospitalet; Department of Cardiology, Copenhagen University Hospital Rigshospitalet

**Keywords:** Atrial Fibrillation (AF), Cardiomyopathy, Genetic variants

## Abstract

Atrial fibrillation (AF) is a common cardiac arrhythmia associated with an increased risk of stroke, heart failure, and death. Recent studies suggest that early-onset AF increases the risk of developing heart failure and dilated cardiomyopathy. This study aims to identify genetic variants in a large set of cardiomyopathy genes among early-onset AF individuals. We conducted targeted sequencing of 29 cardiomyopathy-associated genes in 478 individuals from a Danish cohort with early AF onset. Additionally, we analyzed whole exome sequencing data from 374,289 individuals from the UK Biobank, including 29,108 individuals with AF. 8.8% of the Danish individuals with early-onset AF carry truncating variants in cardiomyopathy-associated genes. The prevalence of truncating variants in the UK Biobank analysis ranges from 3.8% in the group with early AF onset to 1.4% in the group without AF diagnosis. This suggests that genetic testing for cardiomyopathy could be relevant in selected individuals with early AF diagnosis.

## 1. Introduction

With a lifetime risk of up to 30%(1), atrial fibrillation (AF) is the most common cardiac arrhythmia, and represents a serious burden for patients and healthcare systems(2). Among individuals with AF, one in five suffers a stroke, and two in five develop heart failure (HF)(1), leading to increased risk of morbidity and mortality.

While AF has been extensively studied, its association with subsequent cardiovascular diseases and mortality across age groups is not entirely understood. A recent study found that the risk of cardiomyopathy and HF was highest among those with AF diagnosis early in life(3). Moreover, an earlier onset of AF was associated with a considerable decrease in life expectancy compared with a later onset of the disease(3). It has been suggested to be due to a shared genetic architecture of AF and cardiomyopathy(3). Atrial cardiomyopathy has been suggested as both an important component in AF pathogenesis, and as a potential precursor to more severe, ventricular cardiomyopathy phenotypes(4, 5).

In a cohort of 1,293 individuals with AF before age 65, Yoneda et al. reported an increased prevalence of potentially pathogenic genetic variants in genes associated with arrhythmia syndromes, cardiac structure, and cardiomyopathy(6). Individuals who were carriers of such variants also exhibited an increased mortality compared to non-carriers(7). In line with these findings, the newest AF guidelines from the American College of Cardiology and the American Heart Association now recommend that genetic testing could be considered in early-onset AF patients without obvious risk factors(8). However, the prevalence of cardiomyopathy-associated genetic variants in early-onset AF in large population-based studies has not been thoroughly explored.

This study included next-generation sequencing data from Danish AF patients diagnosed before the age of 45 without known cardiomyopathy and HF, and whole exome sequencing data from 374,289 individuals from the UK Biobank. Focusing on pathogenic variants in well-established cardiomyopathy genes, this study aimed to provide new insights into the burden of rare variants in cardiomyopathy-associated genes according to age of AF onset.

## 2. Methods

The study followed the *Strengthening the Reporting of Observational Studies in Epidemiology* (STROBE) reporting guideline.

### 2.1 Danish early-onset atrial fibrillation cohort

Individuals of European ancestry diagnosed with AF before the age of 45, and without a prior history of cardiomyopathy or HF, were identified and recruited using the Danish National Patient Registry(9). DNA was extracted from leukocytes in peripheral blood samples. Following DNA fragmentation with endonucleases, hybridized molecules were created using gene specific probes from the Illumina TruSight Cardio sequencing kit (Illumina Sequencing, USA) and captured using magnetic beads. Following PCR amplification, sequencing was conducted using Illumina HiSeq 2500 and NextSeq technology. Sequenced reads were trimmed and filtered for low-quality reads, aligned to the Human Reference Genome (build GRCh37/bg19) using the Burrows-Wheeler Aligner software(10), and postprocessed according to the Genome Analysis Toolkit best practice guidelines(11). The sequencing, quality control, and bioinformatic post-processing pipeline have previously been described in detail by Ahlberg et al(12).

This cohort has previously been used for studies of the implications of truncating variants in *TTN*(12) and *RBM20*(4) in early-onset AF.

### 2.2 UK Biobank cohort

The UK Biobank (https://www.ukbiobank.ac.uk/) is a large-scale biomedical database and research resource comprised of clinical and genetic data on over 500,000 individuals, aged between 40 and 69 years at inclusion. Participants were enrolled from across the United Kingdom between 2006 and 2010(13, 14).

Phenotypes were defined by the *International Classification of Diseases, 10th revision* (ICD-10) and have been described in **Supplementary Table S1**. The diagnosis of AF in the UK Biobank was based on medical records from hospitals and primary care visits. Participants without a registered AF diagnosis were considered as the reference group. The AF group was further stratified by age of onset into four subgroups: onset below age 45, between ages 45 and 54, between ages 55 and 64, and at age 65 or older. Participants with a pre-existing cardiomyopathy diagnosis, either at the time of inclusion or prior to AF diagnosis, were excluded from the study.

We extracted genetic variants from the UK Biobank whole exome sequencing dataset, which included 404,010 participants of European ancestry. The exome sequencing methodology, comprising quality control, alignment, variant calling, and annotation, has been described in detail previously(15). For this analysis, we considered only variant sites with at least 90% genotype coverage and a read depth greater than ten. Samples were filtered based on heterozygosity, missing rates, excess relativeness, and kinship inference as per UK Biobank resource 531. Additionally, samples showing discrepancies between self-reported sex and genetically determined sex were excluded. Ultimately, 374,289 individuals met the criteria for inclusion in the analysis.

A detailed flowchart of the participant selection for the study cohort and subsequent analyses is shown in **Supplementary Figure S1**.

### 2.3 Candidate gene and variant selection

We focused on genetic variation in 29 well-established cardiomyopathy genes, based on guidelines outlined by the American College of Medical Genetics and Genomics (ACMG)(16) from 2018. Minor adjustments were made based on recent studies conducted in 2021 and 2022, which provided updated information on genes strongly or moderately associated with dilated cardiomyopathy (DCM)(17), hypertrophic cardiomyopathy (HCM)(18), and arrhythmogenic right ventricular cardiomyopathy (ARVC) (19).

For DCM, we included twelve genes associated with the disease: *BAG3*, *DES*, *DSP*, *FLNC*, *LMNA*, *MYH7*, *PLN*, *RBM20*, *SCN5A*, *TNNC1*, *TNNT2*, and *TTN*. To minimize noise from *TTN* isoforms not predominantly expressed in cardiac tissue, we focused on the constitutively expressed exons, which represent exons spliced in at a rate of over 90% (*TTN*-PSI90) (20).

In HCM, we examined *MYH7* and *MYBPC3* due to the well-described pathogenicity of variants in these genes, as well as other genes with strong evidence for HCM, such as *ALPK3*, *MYH6*, *PTPN11* and *TTR*. For ARVC, we selected *PKP2*, *DSG2*, *DSP*, *DSC2*, *JUP*, *TMEM43*, and *PLN*, all of which are linked to desmosomal diseases. We excluded *RYR2* as a candidate gene, as its variants typically cause catecholaminergic polymorphic ventricular tachycardia (CPVT) rather than ARVC(19).

**Figure 1:**
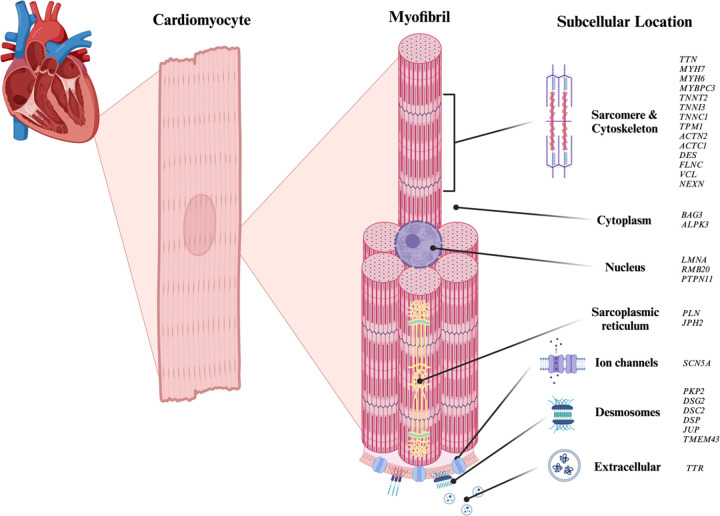
Subcellular localization of selected genes in cardiomyocytes. This figure illustrates selected genes involved in cardiomyopathy, categorized by their protein product distribution in different subcellular locations within a cardiomyocyte. The genes are represented according to their known or predicted functional roles in various cellular compartments, providing insights into their contributions to cardiomyocyte biology and disease mechanisms.

The association between these genes and each of the three cardiomyopathy subtypes listed above, including those with strong and moderate significance, is detailed in **Supplementary Figure S2**. For a more in-depth description of each gene and its associated cellular location within the cardiomyocyte, refer to Fig. 1.

In the main analysis, we prioritized rare variants (minor allele frequency [MAF] < 1%) predicted to lead to loss of function (pLOF variants). These were defined as variants leading to gain or loss of a stop codon, frameshifts, or variants in canonical splice sites. Variant annotation was performed using dbSNP(21) (version 4.1a) and SnpEff(22) (version 5.0). Splice site variants were annotated using SpliceAI(23), and only included variants with a SpliceAI score > 0.8. Furthermore, this analysis assumes that all truncating variants identified have the same impact on the functionality of their respective proteins.

We measured the prevalence of genetic variants in each group by calculating the percentage of individuals with at least one genetic variant. We plotted the prevalence according to the different types of cardiomyopathy. In cases where a gene was associated with more than one type of cardiomyopathy, we represented the prevalence in a chord diagram by dividing and distributing it between the relevant cardiomyopathy classes (*Circlize* Package version 0.4.16 in R version 4.1.0(24)).

### 2.4 Secondary analysis on damaging missense variants

As a secondary analysis, we examined rare missense variants (MAF < 1%) in the UK Biobank. Pathogenicity was assessed using the Missense Tolerance Ratio (MTR), which measures the ratio of observed to expected missense variants within a 31-amino-acid sliding codon window. MTR values were obtained from the Million Exome Variant Browser, developed by the Regeneron Genetics Center (RGC-EM)(25).

This dataset includes genetic variation from 985,830 individuals, obtained from the combination of multiple biobanks such as gnomAD and TOPMed. We focused on the lowest 5th percentile of MTR sites, representing the most constrained regions of the gene. These regions have been shown to be significantly enriched with pathogenic missense variants when compared to ClinVar(25).

### 2.5 Statistical analysis

In the UK Biobank cohort, we investigated the distribution of deleterious variants across cardiomyopathy genes among individuals with AF. We defined carriers as those individuals who carried at least one variant in the aforementioned genes. We estimated odds ratios (OR) for carrying a rare variant according to age of AF onset using multivariable logistic regressions, adjusted for sex, age at inclusion, and six first genotyped principal components. Statistical significance was defined as a two-tailed p-value < 0.05. All statistical analyses were conducted in R version 4.1.0(24).

## 3. Results

This study included 478 Danish individuals and 374,289 participants from the UK Biobank. Baseline characteristics of the cohorts are summarized in Table 1. In the Danish cohort, all individuals had an AF diagnosis at time of inclusion, with a median (1st -3rd quartile) age at AF diagnosis of 28 (23–34) years. Among them, 85 participants (17.8%) were female. In contrast, the UK Biobank included 29,108 individuals diagnosed with AF and 345,181 participants without an AF diagnosis, who served as controls. The median (1st -3rd quartile) age at enrollment in the UK Biobank was 58 (51–63) years, and 201,335 participants (53.8%) were female.

### 3.1 High prevalence of truncating variants in the Danish cohort

**Figure 2:**
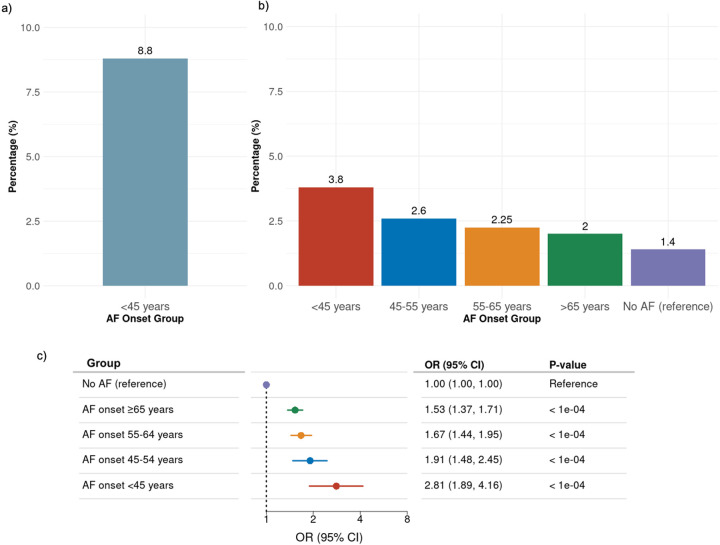
Percentage and odds ratio of participants carrying predicted Loss-of-Function (pLOF) variants in cardiomyopathy genes based on age at atrial fibrillation (AF) diagnosis. **a:** Danish cohort data showing the percentage of carriers. **b:** UK Biobank data stratified by age at AF onset. The highest prevalence of carriers is seen in individuals with AF onset before 45 years (red), while the lowest is seen in individuals with late-onset AF (green) or no diagnosis (purple). **c:** Forest plot of UK Biobank data, displaying odds ratios (ORs), confidence intervals (CI) and p-values of carrying truncating variants, categorized by age at AF onset. Each group is compared to the reference group for statistical significance.

Among 478 Danish individuals diagnosed with AF before age 45 and no known cardiovascular comorbidities, 42 individuals (8.8%) were carriers of 38 rare truncating variants in well-established cardiomyopathy-related genes (Fig. 2A). This cohort exhibited genetic variants in nine of the 29 selected genes.

Of the 38 variants identified, 26 were classified as variants of unknown significance (VUS), while the remaining variants were categorized as pathogenic (five variants), pathogenic/likely pathogenic (six variants), and likely pathogenic (one variant), according to ClinVar(25).

The *TTN* gene was the most frequently affected, with 29 (69.0%) of the 42 individuals harboring a truncating variant (see **Supplementary Table S3**). *TTN* truncating variants accounted for the majority (n = 25, 65.8%) of all pLOF identified in the cohort. *LMNA* followed, with truncating variants observed in three individuals (7.9%), as shown in **Supplementary Table S3**. Furthermore, truncating variants were identified in two individuals for *RBM20*, *TPM1*, and *DSC2* (5.3% per gene), respectively, whereas *MYBPC3*, *PKP2*, *MYH7*, and *SCN5A* had variants in a single individual each (2.6% per gene). The distribution of disease-associated variants in this cohort revealed that 88.1% of all variants were associated with DCM genes (e.g., *TTN*, *LMNA*, *SCN5A, RBM20, MYH7 and TPM1*), 3.9% to HCM genes (*MYBPC3* and *MYH7*), and 7.9% to ARVC genes (*PKP2* and *DSC2*).

### 3.2 Rare pLOF variants in the UK Biobank

A total of 634 individuals (2.17%) from the AF-diagnosed population in the UK Biobank were identified as carriers of rare truncating variants in established cardiomyopathy genes. We observed an inverse dose-response relationship between the age of AF onset and the prevalence of rare pLOF variants, as shown in Fig. 2B. The highest prevalence of carriers was found among individuals diagnosed with AF below the age 45, at 3.8%. In comparison, the prevalence was 2.6% for those with AF onset between 45 and 54 years, 2.2% for 55 to 64 years, 2.0% for those aged 65 and older, and 1.4% among individuals without an AF diagnosis.

Details on the truncating variant carrier counts are presented in **Supplementary Table S4**, where we report the variant count for AF patients with onset < 45 years, the overall AF population, and the entire study population from UK Biobank. Additionally, percentages are reported based on the size of each group, providing a comparative distribution of truncating variants for the 29 selected genes across different cohorts.

Individuals with early-onset AF had a significantly higher OR for carrying a pLOF variant (Fig. 2C). Compared with referents without AF, there was a 2.81-fold increase in OR for carrying a pLOF variant in individuals with AF onset before 45 years of age (95% CI 1.89–4.16; P < 0.0001), representing the highest risk. This was followed by a 1.91-fold increase in individuals with onset between 45 and 54 years (95% CI 1.48–2.45; P < 0.0001), a 1.67-fold increase with onset between 55 and 64 years (95% CI 1.44–1.95; P < 0.0001), and a 1.53-fold increase with onset at or above 65 years (95% CI 1.37–1.71; P < 0.0001).

**Figure 3:**
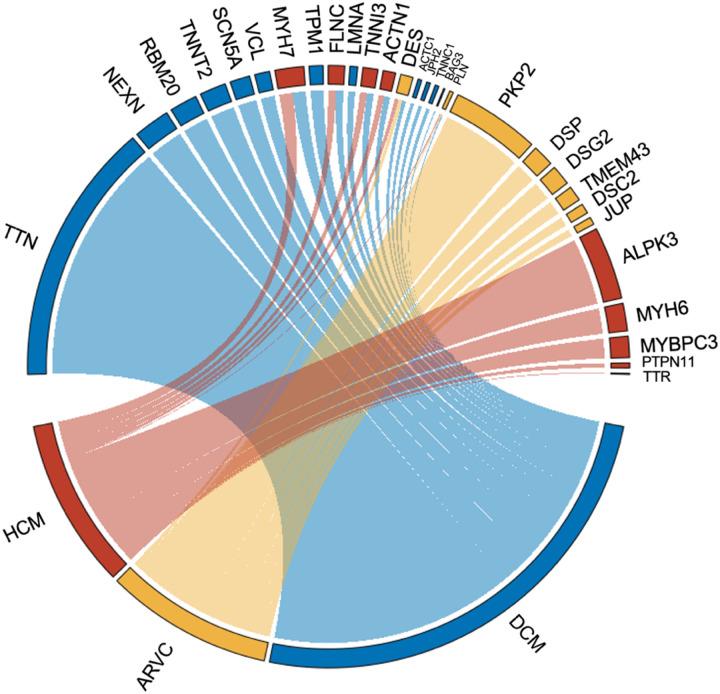
Distribution of rare predicted Loss-of-Function (pLOF) variants in individuals diagnosed with atrial fibrillation (AF) according to genes and their association with cardiomyopathy. Chord diagram illustrating the distribution of rare pLOF variants according to genes and corresponding cardiomyopathy subtypes. The blue segments represent variants in genes associated with dilated cardiomyopathy (DCM), the red segments correspond to variants in hypertrophic cardiomyopathy (HCM)-associated genes, and the yellow segments indicate variants in genes associated with arrhythmogenic right ventricular cardiomyopathy (ARVC).

Furthermore, we examined the distribution of variants across the 29 investigated cardiomyopathy genes in the UK Biobank (Fig. 3), and found that genes associated with DCM represented 57% of all variants present in the AF group in the UK Biobank, followed by 22% of variants related to ARVC and 21% to HCM. The majority of the truncating variants were found in *TTN*, representing 32.8% of all rare truncating variants identified in the AF population, followed by variants in *PKP2* (10.9%) and *ALPK3* (9.1%).

### 3.3 Missense Variants in the UK Biobank

We identified 389 individuals with AF (1.33%) from the AF-diagnosed population as carriers of rare missense variants (MTR < 5%) in the studied genes. The highest prevalence, 2.19%, was seen in individuals diagnosed below the age of 45 (**Supplementary Figure S3A)**, showing an inverse dose-response-like pattern. The prevalence rates for other age groups were 1.34% for 45 to 54 years, 1.31% for 55 to 64 years, 1.31% for those aged 65 and above, and 1.28% among individuals without an AF diagnosis. **Supplementary Table S5** provides the count of carriers among individuals with AF across age groups.

**Supplementary Figure S3B** illustrates the OR for carrying a missense variant among different age groups of AF onset. Carriers with AF onset before 45 years of age had a 1.71-fold increase in OR for carrying a variant (95% CI 1.02–2.85; P = 0.04), compared with referents without AF. No statistically significant associations were found for groups with later onset of AF.

Of the identified missense variants, 71.8% were in genes associated with HCM, 27.2% were in genes associated with DCM, and 1% were in genes associated with ARVC (**Supplementary Figure S4**). The majority of these variants were identified in the *TTR* gene (54%), followed by *MYH7* (8.9%) and *FLNC* (7.4%).

### 3.4 Aggregated prevalence of genetic variants in the UK Biobank

**Figure 4:**
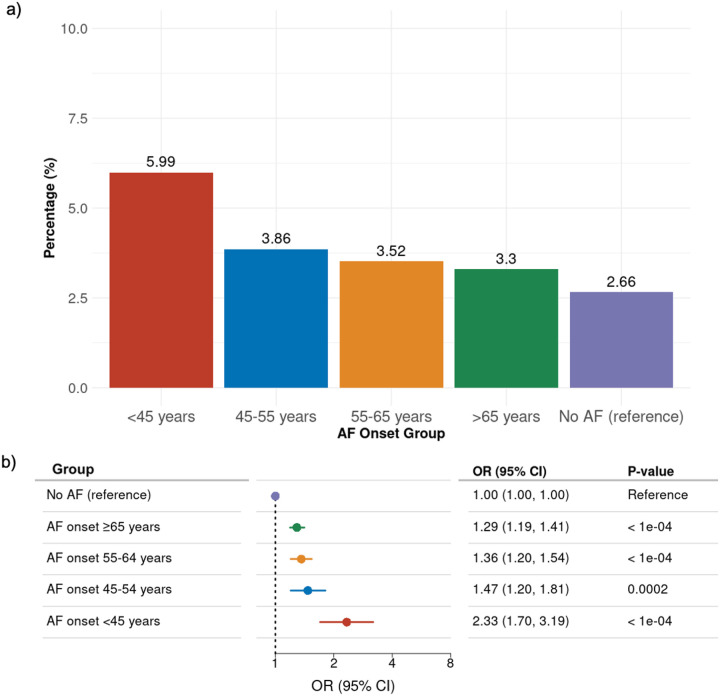
Prevalence and odd ratios of rare missense and predicted Loss-of-Function variants in cardiomyopathy genes according to age at atrial fibrillation diagnosis. **a:** UK Biobank data on rare truncating and missense variant carrier percentages stratified by age of onset. **b:** Forest plot displaying ORs, CI and p-values for the carriers of truncating and missense variants, comparing AF onset groups to the reference population in the UK Biobank.

Integrating both truncating and missense variants indicated a larger aggregated percentage of carriers while following the same pattern. A total of 982 individuals (3.37%) from the AF population carried genetic variants (see **Supplementary Table S6**). Figure 4A shows a consistent pattern, with the highest prevalence seen in early-onset AF individuals at 5.99%. In comparison, the combined prevalence was 3.86% in individuals with AF onset between 45 and 54 years, 3.52% in the 55 to 64 years group, 3.30% in those aged 65 and above, and 2.66% among individuals without an AF diagnosis.

Figure 4B shows the aggregated OR of carrying either rare truncating or missense variants within the UK Biobank population across different age of AF onset groups.

## 4. Discussion

The present study combined data from a cohort of early-onset AF patients with large-scale clinical and genetic information from the UK Biobank, to elucidate the prevalence of genetic variants in known cardiomyopathy genes in patients with AF. We observed that early-onset AF was significantly associated with a greater prevalence of rare genetic variants in the 29 genes associated with either DCM, HCM or ARVC.

Among the Danish individuals with early onset AF, more than one in twelve (8.8%) carried a rare truncating variant. The majority of these variants were in DCM-associated genes, with *TTN* variants accounting for 65.8% of all truncating variants. Nearly two-thirds of those identified were variants of unknown significance. *TTN* encodes titin, a key structural protein in the sarcomere, and truncating variants in this gene are well-established contributors to the development of DCM(26) and AF.

Following *TTN*, *LMNA* was the second gene with the most variants in this cohort, representing 7.9% of the variants found. Variants in *LMNA*, which encodes lamin A/C, are commonly associated with early-onset AF and are linked to severe complications such as DCM and sudden cardiac death(27–29). Additionally, we identified a single nonsense variant in *SCN5A*. While this sodium channel is primarily susceptible to missense variants that are associated with atrioventricular block and DCM(29, 30), the nonsense variant we identified has been classified as pathogenic as it alters intracellular sodium and calcium homeostasis(26). As previously reported(4), we also observed two pLoF variants in *RBM20*, a regulator of splicing for various cardiac genes, including *TTN* and *TPM1*(4). *TPM1*, encoding the actinbinding tropomyosin 1 protein, exhibited two variants in this Danish cohort. Additionally, one individual carried a truncating *MYBPC3* variant, and another individual carried a nonsense variant in *MYH7*. Notably, *MYBPC3* and *MYH7* are known to be involved in 70% of sarcomere gene variant-positive HCM(31).

Furthermore, we identified three variants in two desmosomal genes associated with ARVC, one in *PKP2* and two in *DSC2*. *PKP2* encodes plakophilin 2, a desmosomal protein crucial in ARVC, where it disrupts intracellular calcium handling and predisposes patients to ventricular arrhythmias(26). *DSC2*, a known ARVC disease susceptibility gene, is essential for cardiac desmosome formation, early cardiac morphogenesis, and normal cardiac function(32).

Analyses in the UK Biobank offered insights into the prevalence and distribution of genetic variants across different age groups. Specifically, we found an OR of 2.8 for carrying truncating variants in the individuals with early-onset AF compared to the reference group. Previous studies have described a similar pattern(6). In our study, we focused on constitutive exons with a TTN PSI > 90(33) and selected variants with a MAF of < 1%(34). This approach allows us to focus on rarer, potentially more pathogenic variants that are likely to have a greater impact on disease development.

The majority of these truncating variants were found in key genes associated with DCM (e.g. *TTN* and *RBM20*), HCM (e.g. *ALPK3, MYH6* and *MYBPC3*), and ARVC (e.g. *PKP2, DSP* and *DSG2*). In this cohort, variants in genes associated with DCM accounted for 60% of carriers, driven largely by the high prevalence of *TTN* truncating variants(35–37). Other prominent contributors included well-established DCM-related genes such as *NEXN*(38), *MYH7*(6, 29), *RBM20*(4, 6, 29), *TNNT2*(29) and *SCN5A*(26, 29, 30).

In contrast to our findings from the Danish cohort, *LMNA* variants were relatively rare in the UK Biobank cohort. One possible explanation is that *LMNA* variants typically lead to early-onset DCM(27), often manifesting before the age range of individuals included in the UK Biobank, which may have resulted in the underrepresentation of these variants.

Beyond DCM, 16% of the truncating variant carriers harbored HCM-associated variants in genes including *ALPK3*(39), *MYH7*(40), *MYH6*(18) and *MYBPC3* (41). The majority of variants in HCM genes were in *MYH7*, a sarcomeric gene encoding the b-myosin heavy chain, known to be implicated in both DCM and HCM depending on the penetrance(40, 41), accounted for a significant proportion of carriers.

In total, 24% of UK Biobank participants diagnosed with AF and carriers of genetic variants had truncating variants in genes previously linked to ARVC, with *PKP2* being the most frequent. Additional variants were found in other ARVC-associated genes such as *DSP*, *DSG2*, and *TMEM43*(26), all of which encode desmosomal proteins. Defects in the structure of desmosomes result in cardiac myocyte detachment and cell death, leading to fibrofatty tissue infiltration and electrical instability(42).

To elucidate a more detailed genetic landscape of AF across age groups, we also examined rare missense variants, applying an MTR score to focus on highly conserved regions. While the overall prevalence of missense variants showed a similar trend to that of truncating variants, their impact was less pronounced, with early-onset AF patients having a 1.7-fold increased OR of carrying these variants compared to the reference group. Missense variants were predominantly found in genes associated with HCM, accounting for 71% of carriers. *MYH7* had the highest prevalence of missense variants in this population, emphasizing the relevance of both missense and truncating variants in *MYH*7.

A higher prevalence of rare missense variants was observed in the *TTR* gene compared to truncating variants. *TTR* encodes the protein transthyretin, and mutations in this gene have been associated with cardiac amyloidosis (ATTR-CA) and HCM(43). Another significant gene in the missense carrier group was *FLNC*. A previous study showed that *FLNC* missense variants were predominantly associated with HCM, while truncating variants were rather strongly associated with DCM(44).

The analysis of missense variants across cardiomyopathies revealed notable differences compared to truncating variants. Truncating variants were most frequently found in genes associated with DCM, whereas HCM and ARVC genes had a similar prevalence of missense and truncating variants. In contrast, disease-associated missense variants were most commonly found in HCM genes, followed by DCM genes, while missense variants in ARVC-associated genes were rare. These findings hint at distinct genetic mechanisms underlying each variant type and their respective roles in specific cardiomyopathies.

In summary, our findings indicate that individuals with very early onset of AF have a high prevalence of potentially pathogenic variants in cardiomyopathy-associated genes. Thus, these individuals may benefit from more thorough clinical monitoring to aid in identifying individuals at an elevated risk of developing cardiomyopathy and subsequent HF. While systematic genetic screening for rare variants is unlikely to be implemented in the near future, incorporating genetic data into clinical practice could improve cardiomyopathy risk stratification in individuals with AF.

### 4.1 Limitations

The findings from this study should be interpreted with the following limitations in mind. First, the Danish cohort only included individuals with very early-onset AF, which is not representative of the typical patient with AF. This selection may partly explain the higher prevalence of truncating variants observed in the Danish cohort compared to individuals with early-onset AF in the UK Biobank. Additionally, a healthy-volunteer bias in the UK Biobank may lead to an underrepresentation of individuals with pathogenic genetic variants, potentially limiting the generalizability of these findings. Furthermore, targeted sequencing of the Danish cohort did not include *ALPK3*, *ACTC1*, *TNNC1*, and *PLN*, which may affect the comprehensiveness of our variant analysis.

Moreover, not all variants may be equally pathogenic or penetrant. As participants had to survive until inclusion in the respective cohorts, this could introduce immortal time bias and result in the underrepresentation of certain variants compared to others. Finally, both cohorts were of white European ancestry, and it cannot be assumed that the findings can be generalized to other population groups.

### 4.2 Conclusion

By using two different AF cohorts, we found that a significant number of individuals with early onset of AF carried rare pathogenic variants in hallmark cardiomyopathy genes. Prevalence of truncating variants ranged from one in twelve, to one in fifteen of individuals with AF onset below age 45. These findings support that screening and clinical surveillance for cardiomyopathy might be relevant for individuals with early-onset AF.

## Supplementary Material

Supplementary Files

This is a list of supplementary files associated with this preprint. Click to download.

- EJHGSupplementalMaterials.pdf

## Figures and Tables

**Table 1:** Baseline characteristics at inclusion in the Danish and UK Biobank cohorts.

	Danish cohort (N = 478)	UK Biobank (N = 374,289)				
	AF onset < 45 years	No AF (reference)	AF onset < 45 years	AF onset 45–54 years	AF onset 55–64 years	AF onset > 65 years
N	478	345,181	684	2,464	7,775	18,185
Sex						
Female, n (%)	85 (17.8)	190,832 (55.3)	207 (30.3)	715 (29.0)	2,534 (32.6)	7,047 (38.8)
Male, n (%)	393 (82.2)	154,349 (44.7)	477 (69.7)	1,749 (71.0)	5.241 (67.4)	11,138 (61.2)
Age at AF diagnosis, years, median (1st -3rd quartile)yx	28 (23, 34)	No AF	40 (36, 43)	51 (49, 53)	61 (58, 63)	71 (68, 75)
BMI, kg/m^2^, median (1st -3rd quartile)	26.0 (23.5, 29.0)	26.6 (24.1, 29.7)	27.6 (24.8, 31.2)	28.2 (25.3, 31.9)	28.3 (25.4, 32.1)	28.1 (25.3, 31.5)
Blood pressure						
Systolic, mmHg, median (1st - 3rd quartile)	128 (120, 135)	138 (126, 152)	133 (122, 146)	137 (124, 150)	140 (127, 153)	146 (134, 160)
Diastolic, mmHg, median (1st -3rd quartile)	79 (71, 83)	82 (75, 89)	81 (75, 89)	83 (75, 91)	83 (75, 90.8)	82 (75, 90)
Hypertension, n (%)	30 (6.3)	366 (53.5)	1,488 (60.4)	5,272 (67.8)	13,512 (74.3)	124,154 (36.0)
Diabetes, n (%)	6 (1.3)	27,428 (7.9)	115 (16.8)	503 (20.4)	1,650 (21.2)	4,024 (22.12)
AF, Atrial Fibrillation;						
BMI, Body Mass Index						

## Data Availability

Bona-fide researchers can apply for access to the UK Biobank through https://www.ukbiobank.ac.uk/use-our-data/apply-for-access/. Due to GDPR regulation, access to the Danish National Patient Registry data is not available.
